# Depletion of skeletal muscle satellite cells attenuates pathology in muscular dystrophy

**DOI:** 10.1038/s41467-022-30619-7

**Published:** 2022-05-26

**Authors:** Justin G. Boyer, Jiuzhou Huo, Sarah Han, Julian R. Havens, Vikram Prasad, Brian L. Lin, David A. Kass, Taejeong Song, Sakthivel Sadayappan, Ramzi J. Khairallah, Christopher W. Ward, Jeffery D. Molkentin

**Affiliations:** 1 Department of Pediatrics, Cincinnati Children’s Hospital Medical Center, Cincinnati, OH 45229 USA; 2 Department of Pediatrics, University of Cincinnati, Cincinnati, OH 45229 USA; 3 Division of Cardiology, Johns Hopkins Medical Institutions, Baltimore, MD 21205 USA; 4 Division of Cardiovascular Health and Disease, Department of Internal Medicine, University of Cincinnati, Cincinnati, OH 45267 USA; 5 Myologica, LLC, New Market, MD 21774 USA; 6 Department of Orthopedics and Center for Biomedical Engineering and Technology (BioMET), University of Maryland School of Medicine, Baltimore, MD USA

**Keywords:** Mechanisms of disease, Molecular medicine, Skeletal muscle

## Abstract

Skeletal muscle can repair and regenerate due to resident stem cells known as satellite cells. The muscular dystrophies are progressive muscle wasting diseases underscored by chronic muscle damage that is continually repaired by satellite cell-driven regeneration. Here we generate a genetic strategy to mediate satellite cell ablation in dystrophic mouse models to investigate how satellite cells impact disease trajectory. Unexpectedly, we observe that depletion of satellite cells reduces dystrophic disease features, with improved histopathology, enhanced sarcolemmal stability and augmented muscle performance. Mechanistically, we demonstrate that satellite cells initiate expression of the myogenic transcription factor MyoD, which then induces re-expression of fetal genes in the myofibers that destabilize the sarcolemma. Indeed, MyoD re-expression in wildtype adult skeletal muscle reduces membrane stability and promotes histopathology, while MyoD inhibition in a mouse model of muscular dystrophy improved membrane stability. Taken together these observations suggest that satellite cell activation and the fetal gene program is maladaptive in chronic dystrophic skeletal muscle.

## Abstract (web summary)

Boyer et al. created genetic mouse models of muscular dystrophy in which satellite cells were selectively depleted. The depletion of satellite cells at select times was protective. Myofibers no longer had plasma membrane instability leading to tissue wasting in the muscular dystrophies.

## Introduction

Intrinsic to skeletal muscle are resident progenitor “satellite” cells^1^ essential for the repair of damaged muscle fibers or their replacement following fiber death. Therapeutic strategies towards increasing satellite cell activity or numbers are being actively pursued for treatment of the Muscular Dystrophies (MD), a family of diseases whereby diverse genetic mutations predispose muscle fibers to damage and necrotic death.

While strategies that augment the activity of satellite cells have been widely pursued as a treatment approach in the MDs, the opposite effect has been noted such that reduced satellite cell activity/number could improve or delay histopathology^2^. Similarly, deletion of the transcription factor *Nfix* that underlies fetal myogenic gene expression, resulted in delayed regeneration^3^, and improved pathological hallmarks in dystrophic mice^4^. Here we directly assessed this discrepancy on the contribution of satellite cells to MD pathogenesis in the murine models of Duchenne MD (DMD, *mdx*[mutation in dystrophin gene]) and Limb girdle MD 2F (*Sgcd*^*−/−*^, delta-sarcoglycan gene]) which are devastating and life-limiting MD’s with no cure.

Using developed mouse models with profound satellite cell depletion on the *mdx* and *Sgcd*^*−/−*^ MD mouse genetic backgrounds we show improved MD histopathology, muscle performance, and reduction in muscle susceptibility to contraction injury when satellite cells are absent. Mechanistically, we show that chronic satellite cell activity in MD induces the re-expression of the myogenic transcription factor MyoD and the fetal gene program in the muscle fiber, which destabilizes the sarcolemma and predisposes to muscle damage. Moreover, inhibition of MyoD is partially protective in the *Sgcd*^*−/−*^ MD model, while MyoD re-expression in wild-type (Wt) adult skeletal muscle recapitulates MD disease features. Collectively, the results suggest that chronic satellite cell activation in MD is maladaptive and that limiting the activity of these cells, and the membrane destabilizing aspects of the fetal gene program, may be an effective strategy to mitigate MD pathology.

## Results

### Erk1/2 are required for satellite cell viability upon activation

**Fig. 1:**
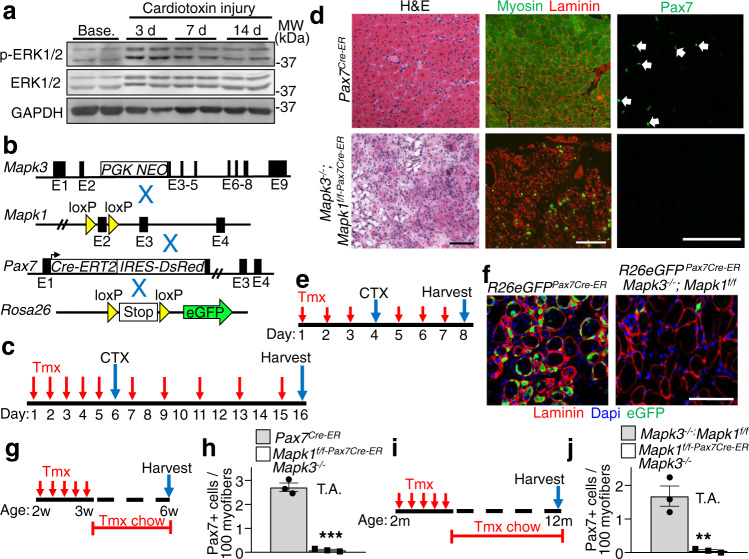
Erk1/2 are required for satellite cell viability upon activation. **a** Western blot analysis for the indicated proteins using tibialis anterior (TA) lysate from samples taken at baseline (Base.) and following 3, 7 and 14 days (d) after cardiotoxin (CTX) administration. Results from two different mice are shown. **b** Schematic representation of breeding of *Mapk3*^*−/−*^ and *Mapk1*-loxP(f)-targeted mice with the *Pax7*^*Cre-ERT2*^ mouse as well as with the Cre-dependent *R26eGFP* reporter mouse. **c** Schematic showing the timing of tamoxifen (Tmx) and CTX treatments in 2-month-old mice. **d** Representative images of TA muscle sections from mice of the indicated genotypes stained with H&E (left panels), anti-myosin antibody (MF-20, green) and anti-laminin antibody (red, middle panels) or with anti-Pax7 antibody (green, right panels) 10 days post-CTX injury. Scale bars = 100 μm. Samples from 3 different mice were analyzed. **e** Experimental scheme showing mice injected with Tmx for 3 days, then with CTX on day 4, followed by 3 more days of Tmx injections. **f** Representative histological sections showing eGFP fluorescence (green) and immunostained for laminin (red) 4 days post-CTX injection in mice of the indicated genotypes. Nuclei are stained with Dapi (blue). Scale bar = 100 μm. Samples from 3 different mice were analyzed. **g** Schematic representation of the Tmx treatment regimen. Two-week-old mice received Tmx injections for 5 consecutive days and were placed on Tmx chow after weaning. **h** Quantification of Pax7^+^ satellite cells from uninjured TA muscle sections from mice of the indicated genotypes. *n* = 3 mice per group. Significance was determined using a two-tailed Student’s *t* test, ***P < 0.001. **i** Schematic representation of Tmx treatment. Two-month-old mice received daily Tmx injection for 5 consecutive days and were subsequently placed on Tmx chow for 10 months. **j** Quantification of satellite cell numbers from uninjured TA muscle sections from 1-year-old mice of the indicated genotypes. *n* = 3 mice per group. Significance was determined using a two-tailed Student’s *t* test, **P = 0.005. Data represent mean ± SEM for all graphs.

Here we identified a mouse model to selectively delete satellite cells through genetic loss of a critical survival signaling pathway involving extracellular signal-regulated kinases 1/2 (Erk1/2)^5,6^. With respect to skeletal muscle, Erk1/2 expression increased on days 3 and 7 of regeneration in the tibialis anterior (TA) muscle following acute cardiotoxin injury, with a leveling of expression by day 14 when regeneration was complete (Fig. 1a). To examine the functional role of these kinases in muscle regeneration we crossed *Mapk3*^*−/−*^ (Erk1 protein)^7^ and *Mapk1-loxP* site (fl) targeted mice (Erk2 protein)^8^ together with the tamoxifen inducible *Pax7*^*Cre-ERT2*^ mouse^9^ to permit satellite cell-specific ablation (Fig. 1b). We then treated eight-week-old *Mapk3*^*−/−*^; *Mapk1*^*f/f-Pax7Cre-ER*^ mice with tamoxifen and harvested muscles ten days after cardiotoxin injection (Fig. 1c). TA muscles from *Mapk3*^*−/−*^; *Mapk1*^*f/f-Pax7Cre-ER*^ mice were completely devoid of newly regenerating myofibers as demonstrated by hematoxylin and eosin (H&E) staining and by immunostaining for myosin heavy chain (Fig. 1d, green). Immunohistochemistry for Pax7 expression (satellite cell marker) was also absent in TA muscle ten days after cardiotoxin injury in *Mapk3*^*−/−*^; *Mapk1*^*f/f-Pax7Cre-ER*^ mice but not Pax7Cre-ER controls (Fig. 1d). These results suggest that Erk1/2 are required in satellite cells for their survival and muscle regeneration.

*Mapk3*^*−/−*^; *Mapk1*^*f/f-Pax7Cre-ER*^ mice were also crossed with the *Rosa26eGFP*^10^ Cre-dependent reporter mouse line to directly track satellite cells in vivo (Fig. 1b). Eight-week-old *R26eGFP* ^*Pax7Cre-*ER^; *Mapk3*^*−/−*^*; Mapk1*^*f/f*^ mice were treated with tamoxifen to delete *Mapk1*, followed by cardiotoxin administration and the TA muscle was harvested four days later (Fig. 1e). Immunohistochemistry essentially showed an absence of eGFP labeled satellite cells in the TA muscles of *R26eGFP* ^*Pax7Cre-*ER^; *Mapk3*^*−/−*^*; Mapk1*^*f/f*^ mice compared with abundant Pax7^+^ eGFP cells in control muscle four days post cardiotoxin injury (Fig. 1f). These data suggest that activated Erk1/2-deficient satellite cells die and are cleared following acute injury.

To assess the need for Erk1/2 in satellite cells at baseline (uninjured muscles), *Mapk3*^*−/−*^; *Mapk1*^*f/f-Pax7Cre-ER*^ and control mice were treated with tamoxifen beginning at two weeks of age, a time when satellite cells are proliferating and actively contributing to developmental muscle growth^11,12^. Tamoxifen treatment resulted in a 96% decrease in satellite cells in six-week-old *Mapk3*^*−/−*^; *Mapk1*^*f/f-Pax7Cre-ER*^ mice (Fig. 1g, h). Long-term analyses showed depletion (97% decrease) of the satellite cell pool in twelve-month-old *Mapk3*^*−/−*^; *Mapk1*^*f/f-Pax7Cre-ER*^ mice treated with tamoxifen starting at two months of age (Fig. 1i, j). The observation that mice could survive 12 months without satellite cells also suggests that existing muscle is stable when injury is absent. Singular deletion of *Mapk3* or *Mapk1* (using the LoxP-Cre strategy) did not affect satellite cell viability suggesting that both genes must be deleted to lose satellite cells.

Satellite cells are in quiescence in mature skeletal muscle fibers and activated upon injury^11^. Consistent with this, treatment of 6-8-month-old *Mapk3*^−/−^; *Mapk1*^*f/f-Pax7Cre-ER*^ with tamoxifen for four weeks showed no satellite cell loss in the TA muscle compared with controls (Supplementary Fig. 1a–c). In contrast, we show that cardiotoxin injury activated satellite cells in *Mapk3*^*−/−*^; *Mapk1*^*f/f-Pax7Cre-ER*^ mice, leading to satellite cell depletion and loss of myofiber regeneration (Supplementary Fig. 1d).

### Loss of satellite cells reduces muscle damage in a mouse model of LGMD2F

**Fig. 2:**
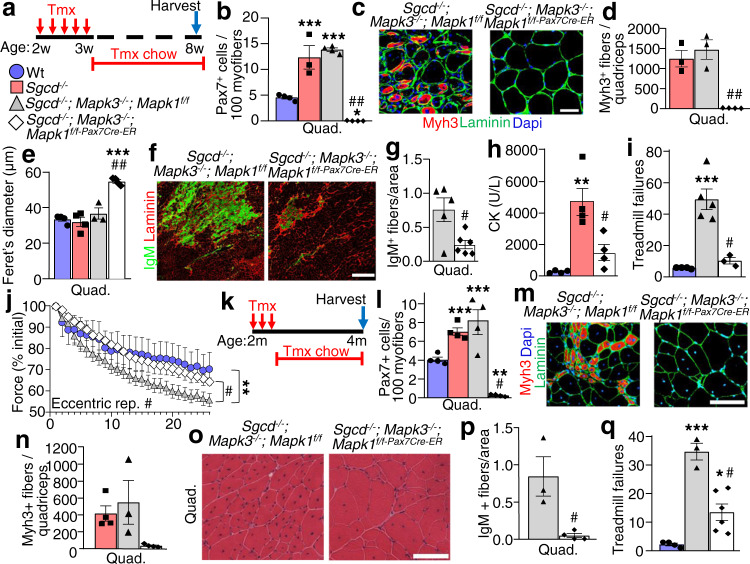
Loss of satellite cells reduces muscle damage in *Sgcd*^*−/−*^ mice. **a** Schematic showing 2-week-old mice receiving a daily Tmx injection for 5 consecutive days and then placed on Tmx chow after weaning until 8 weeks, which applies to all the data in panels **b**–**j**. **b** Quantification of Pax7^+^ satellite cells in muscle sections from the quadriceps (Quad) of mice with the indicated genotypes. *n* = 3 mice for *Sgcd*^*−/−*^ samples and *n* = 4 mice for all other genotypes. One-way ANOVA with Tukey’s multiple comparisons test was used to determine significance, ***P < 0.001 vs Wt, ^##^*P* < 0.001 vs disease controls. **c** Representative quad muscle sections immunostained for Myh3 (red) and laminin (green) in 2-month-old mice of the indicated genotypes. Dapi stained nuclei are in blue. Scale bar = 50 μm. **d** Quantification of the number of Myh3 positive fibers in Quad muscle sections of mice with the indicated genotypes. *n* = 3 mice for *Sgcd*^*−/−*^ and *Sgcd*^*−/−*^; *Mapk3*^*−/−*^; *Mapk1*^*f/f*^ samples and *n* = 4 mice for *Sgcd*^–/–^; *Mapk3*^*−/−*^; *Mapk1*^*f/f-Pax7Cre-ER*^ samples. One-way ANOVA with Tukey’s multiple comparisons test was used to determine significance, ^##^*P* < 0.001 vs disease controls. **e** Average minimal feret’s diameter of myofibers measured from muscle sections of the Quad in mice with the indicated genotypes. *n* = 4 Wt mice; 4 *Sgcd*^*−/−*^ mice; 3 *Sgcd*^*−/−*^; *Mapk3*^*−/−*^; *Mapk1*^*f/f*^; mice and 4 *Sgcd*^*−/−*^; *Mapk3*^*−/−*^; *Mapk1*^*f/f-Pax7Cre-ER*^ mice. A 1-way ANOVA with Tukey’s multiple comparisons test was used to determine significance, ***P < 0.001 vs Wt, ^##^*P* < 0.001 vs disease controls. **f** Representative histological sections of the Quad muscle immunostained for immunoglobulin M (IgM) (green) and for laminin (Red) in mice of the indicated genotypes. Scale bar = 500 μm. **g** Quantification of IgM positive fibers in Quad muscle sections from mice of the indicated genotypes. *n* = 5 mice, *Sgcd*^*−/−*^; *Mapk3*^*−/−*^; *Mapk1*^*f/f*^; *n* = 6 mice, *Sgcd*^*−/−*^; *Mapk3*^*−/−*^; *Mapk1*^*f/f-Pax7Cre-ER*^. Significance was determined using a two-tailed Student’s *t* test, ^#^*P* = 0.01. **h** Average blood creatine kinase (CK) levels from mice of the indicated genotypes at 8 weeks of age. *n* = 4 mice in each group. One-way ANOVA with Tukey’s multiple comparisons test was used to determine significance, and **P < 0.01 vs Wt, ^#^*P* < 0.05 vs *Sgcd*^*−/−*^. **i** Quantification of the number of shock pad visits calculated over 20 min from the start of the protocol. *n* = 4 Wt mice; *n* = 5 *Sgcd*^*−/−*^; *Mapk3*^*−/−*^; *Mapk1*^*f/f*^ mice; *n* = 3 *Sgcd*^*−/−*^; *Mapk3*^*−/−*^; *Mapk1*^*f/f-Pax7Cre-ER*^ mice. A 1-way ANOVA with Tukey’s multiple comparisons test was used to determine significance, ***P < 0.001 vs Wt, ^#^*P* < 0.01 vs *Sgcd*^*−/−*^; *Mapk3*^*−/−*^; *Mapk1*^*f/f*^. **j** Percent change in force transduction for the hindlimb muscles subjected to continuous repetitive eccentric contractions in the 3 genotypes of mice shown based on the key in panel “**a**”. The *x*-axis shows number of progressive repetitive (rep) contractions. A two-way repeated measures ANOVA with a Holm-Sidak post-hoc test was used to determine significance. *n* = 6 Wt mice; *n* = 7 *Sgcd*^*−/−*^; *Mapk3*^*−/−*^; *Mapk1*^*f/f*^ mice; *n* = 4 *Sgcd*^*−/−*^; *Mapk3*^*−/−*^; *Mapk1*^*f/f-Pax7Cre-ER*^ mice. **P < 0.01 vs Wt, ^#^*P* < 0.01 vs disease control. **k** Schematic representation of the Tmx treatment regimen, which applies to all the data in panels **l**–**q**. Two-month-old mice received a daily Tmx injection for 5 consecutive days and were subsequently placed on Tmx chow until 4 months of age. **l** Quantification of Pax7^+^ satellite cells in muscle sections from the Quad of mice with the indicated genotypes. *n* = 4 mice for all groups. A 1-way ANOVA with Tukey’s multiple comparisons test was used to determine significance, **P < 0.01 vs Wt, ***P < 0.01 vs Wt, ^#^*P* < 0.001 vs disease controls. **m** Representative Quad muscle sections immunostained for Myh3 (red) and laminin (green) in 4-month-old mice of the indicated genotypes. Dapi stained nuclei are in blue. Scale bar = 100 μm. ***n*** Quantification of the number of Myh3 positive fibers in Quad muscle sections of mice with the indicated genotypes. *n* = 4 *Sgcd*^*−/−*^ and *Sgcd*^–/–^; *Mapk3*^*−/−*^ and *Mapk1*^*f/f-Pax7Cre-ER*^ mice and *n* = 3 *Sgcd*^*−/−*^; *Mapk3*^*−/−*^; *Mapk1*^*f/f*^ mice. **o** Representative H&E-stained sections of the Quad muscle from 4-month-old mice of the indicated genotypes. Scale bar = 100 μm. **p** Quantification of IgM positive fibers in Quad muscle sections from mice of the indicated genotypes. *n* = 3 *Sgcd*^*−/−*^; *Mapk3*^*−/−*^; *Mapk1*^*f/f*^ mice and *n* = 4 *Sgcd*^*−/−*^; *Mapk3*^*−/−*^; *Mapk1*^*f/f-Pax7Cre-ER*^ mice. Significance was determined using a two-tailed Student’s *t* test, ^#^*P* = 0.01. **q** Quantification of the number of shock pad visits calculated 10 min from the start of the protocol. *n* = 4 Wt mice; *n* = 3 *Sgcd*^*−/−*^; *Mapk3*^*−/−*^; *Mapk1*^*f/f*^ mice and *n* = 6 *Sgcd*^*−/−*^; *Mapk3*^*−/−*^; *Mapk1*^*f/f-Pax7Cre-ER*^ mice. A 1-way ANOVA with Tukey’s multiple comparisons test was used to determine significance, *P < 0.05 vs Wt, ***P < 0.001 vs Wt, ^#^*P* < 0.001 vs *Sgcd*^*−/−*^; *Mapk3*^*−/−*^; *Mapk1*^*f/f*^. Data represent mean ± SEM for all graphs.

Having established a model of satellite cell ablation, we used *Mapk3*^*−/−*^; *Mapk1*^*f/f-Pax7Cre-ER*^ mice to investigate how the absence of satellite cells impacts the pathogenesis of MD. *Mapk3*^*−/−*^; *Mapk1*^*f/f-Pax7Cre-ER*^ mice were crossed onto the *Sgcd*^*−/−*^^13^ background and treated with tamoxifen at two weeks of age followed by analysis six weeks later (Fig. 2a). The three tamoxifen treated control groups were Wt, *Sgcd*^*−/−*^, and Cre-negative *Sgcd*^*−/−*^; *Mapk3*^*−/−*^; *Mapk1*^*f/f*^ mice. Immunostaining for Pax7 in muscles showed a greater than 2-fold expansion of satellite cells in the *Sgcd*^*−/−*^ background compared with Wt, while complete loss of Erk1/2 in *Sgcd*^*−/−*^; *Mapk3*^*−/−*^; *Mapk1*^*f/f-Pax7Cre-ER*^ mice showed a near absence of all satellite cells (Fig. 2b). This loss of satellite cells resulted in the complete lack of regeneration in muscle from *Sgcd*^*−/−*^; *Mapk3*^*−/−*^; *Mapk1*^*f/f-Pax7Cre-ER*^ mice as assessed by Myh3 positivity, and centrally located nuclei in myofibers (Fig. 2c, d and Supplementary Fig. 2a, b). There were also significantly smaller muscles and fewer myofibers in the extensor digitorum longus (EDL) muscle of *Sgcd*^*−/−*^; *Mapk3*^*−/−*^; *Mapk1*^*f/f-Pax7Cre-ER*^ mice counted at eight weeks of age versus the control groups (Supplementary Fig. 2c–e). While myofiber numbers were altered, the muscle that remained in *Sgcd*^*−/−*^; *Mapk3*^*−/−*^; *Mapk1*^*f/f-Pax7Cre-ER*^ mice with satellite cell depletion and harvested at 8 weeks of age showed less histopathology, greater myofiber hypertrophy and less fibrosis compared with the *Sgcd*^*−/−*^, and Cre-negative *Sgcd*^*−/−*^; *Mapk3*^*−/−*^; *Mapk1*^*f/f*^ disease control mice (Fig. 2e and Supplementary Fig. 2c, f, g).

Immunostaining for immunoglobulin M (IgM) protein was also performed as a measure of myofiber sarcolemmal stability, which when present inside a myofiber indicates membrane rupture and MD like disease. Mechanistically, loss of satellite cells in quadriceps from *Sgcd*^*−/−*^; *Mapk3*^*−/−*^; *Mapk1*^*f/f-Pax7Cre-ER*^ mice relative to diseased *Sgcd*^*−/−*^; *Mapk3*^*−/−*^; *Mapk1*^*f/f*^ littermate control mice showed a marked reduction in IgM positive myofibers indicating greater stabilization of the sarcolemma in the absence of satellite cells (Fig. 2f, g), which is also consistent with lower serum creatine kinase (CK) levels in vivo (Fig. 2h).

With evidence of improved muscle quality in satellite cell depleted MD muscle, we next assessed muscle function. Murine models of DMD are sensitive to contraction injury as evidenced by reduced capacity for forced down-hill treadmill running^14^. We show *Sgcd*^*−/−*^ are similarly deficient in down-hill treadmill capacity vs Wt controls (Fig. 2i). However, consistent with the mitigated histopathology seen with satellite cell depetion, *Sgcd*^*−/−*^; *Mapk3*^*−/−*^; *Mapk1*^*f/f-Pax7Cre-ER*^ mice performed significantly better than *Sgcd*^*−/−*^; *Mapk3*^*−/−*^; *Mapk1*^*f/f*^ control mice in downhill running (Fig. 2i). The susceptibility to eccentric contraction induced force loss is a hallmark assay for murine and canine DMD severity and therapeutic efficacy. Remarkably, *Sgcd*^*−/−*^; *Mapk3*^*−/−*^; *Mapk1*^*f/f-Pax7Cre-ER*^ mice lacking satellite cells were protected from eccentric contraction elicited force loss (Fig. 2j), while producing similar levels of normalized force generation.

Next, tamoxifen treatment was initiated in young adulthood (two months of age) to delete satellite cells in *Sgcd*^*−/−*^ mice (Fig. 2k). Quadriceps were then harvested at four months of age, which showed that relative to Wt controls, *Sgcd*^*−/−*^ and *Sgcd*^*−/−*^; *Mapk3*^*−/−*^; *Mapk1*^*f/f*^ disease control mice again had an expansion of satellite cells that is typical of early MD pathogenesis, while *Sgcd*^*−/−*^; *Mapk3*^*−/−*^; *Mapk1*^*f/f-Pax7Cre-ER*^ mice lacking *Mapk1/3* in their satellite cells showed a near complete loss of these cells (Fig. 2l). This adult-specific loss of satellite cells in *Sgcd*^*−/−*^; *Mapk3*^*−/−*^; *Mapk1*^*f/f-Pax7Cre-ER*^ mice was again accompanied by a dramatic reduction in myofiber regeneration as marked by Myh3 positivity, as well as a reduction in tissue histopathology (Fig. 2m–o). While ongoing MD in early adulthood leads to enhanced muscle weights and myofiber hyperplasia, adult satellite cell depleted *Sgcd*^*−/−*^; *Mapk3*^*−/−*^; *Mapk1*^*f/f-Pax7Cre-ER*^ mice failed to show these increases and they were not different from Wt controls (Supplementary Fig. 3a–c). Myofibers were significantly larger in size in muscles from *Sgcd*^*−/−*^; *Mapk3*^*−/−*^; *Mapk1*^*f/f-Pax7Cre-ER*^ mice lacking satellite cells relative to Wt controls and *Sgcd*^*−/−*^ mice with satellite cells (Supplementary Fig. 3d). Mechanistically, there was a significant reduction in IgM positive myofibers in adult satellite cell depleted *Sgcd*^*−/−*^; *Mapk3*^*−/−*^; *Mapk1*^*f/f-Pax7Cre-ER*^ mice versus *Sgcd*^*−/−*^ mice, indicating an enhancement in sarcolemmal stability (Fig. 2p), this enhancement in membrane stability was maintained at least to 7 months of age (Supplementary Fig. 3e, f). Finally, depleting satellite cells between 2-4 months of age also significantly rescued treadmill running performance in *Sgcd*^*−/−*^; *Mapk3*^*−/−*^; *Mapk1*^*f/f-Pax7Cre-ER*^ mice compared to disease controls (Fig. 2q). Thus, the loss of satellite cells in young adult *Sgcd*^*−/−*^ mice protected skeletal muscles from membrane instability and myofiber degeneration and these mice did not show progressive sarcopenia with aging (see discussion).

### Improved histopathology in mdx mice lacking satellite cells

**Fig. 3:**
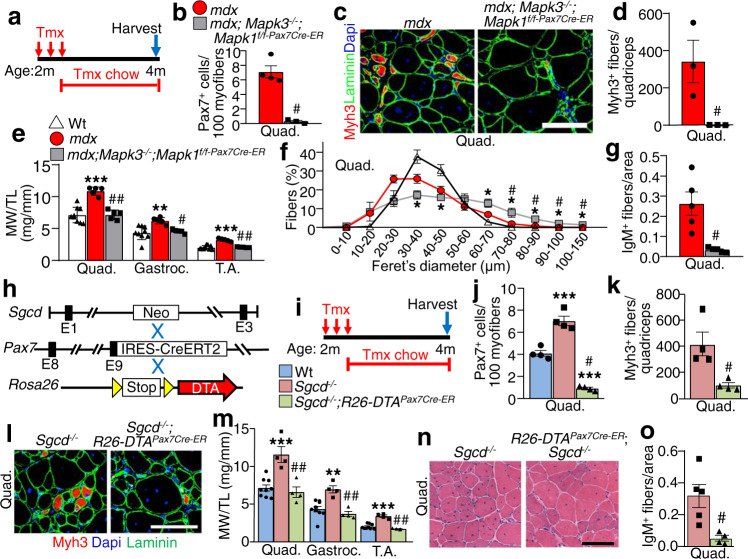
Satellite cell ablation mitigates disease severity in dystrophic mice. **a** Schematic representation of the Tmx treatment regimen in *mdx* mice. Two-month-old mice received daily Tmx injection for 5 days and were subsequently placed on Tmx chow until 4 months of age. **b** Quantification of Pax7^+^ satellite cells from Quad muscle sections from 4-month-old mice of the indicated genotypes. *n* = 4 *mdx* mice and *n* = 3 *mdx*; *Mapk3*^*−/−*^; *Mapk1*^*f/f-Pax7Cre-ER*^ mice. Significance was determined using a two-tailed Student’s *t* test, ^#^*P* = 0.001. **c** Representative Quad muscle sections immunostained for Myh3 (red) and laminin (green) in 4-month-old mice of the indicated genotypes. Dapi stained nuclei are in blue. Scale bar = 100 μm. **d** Quantification of the number of Myh3 positive myofibers in Quad muscle sections of mice with the indicated genotypes. *n* = 3 mice per group. Significance was determined using a two-tailed Student’s *t* test, ^#^*P* = 0.04. **e** Muscle weights (MW) normalized to tibia length (TL) from mice of the indicated genotypes at 4 months of age. *n* = 9 Wt mice and *n* = 5 *mdx* and *mdx*; *Mapk3*^*−/−*^; *Mapk1*^*f/f-Pax7Cre-ER*^ mice. A 1-way ANOVA with Tukey’s multiple comparisons test was used to determine significance, **P < 0.01 vs Wt, ***P < 0.001 vs Wt, ^#^*P* = 0.01 vs *mdx*, ^##^*P* < 0.001 vs *mdx*. The 4-month Wt weight data are also shown in “**m**” and in Supplementary Fig. 3b. **f** Minimal feret’s myofiber diameter distribution from the Quad muscle of 4-month-old mice with the indicated genotypes. *n* = 4 Wt mice; *n* = 3 *mdx* and *mdx*; *Mapk3*^*−/−*^; *Mapk1*^*f/f-Pax7Cre-ER*^ mice. A 1-way ANOVA with Tukey’s multiple comparisons test was used to determine significance, *P < 0.05 vs Wt, ^#^*P* < 0.05 vs *mdx*. The 4-month Wt feret’s diameter data are also shown in Supplementary Fig. 3d. **g** Quantification of IgM positive fibers in quad muscle sections from mice of the indicated genotypes. *n* = 5 mice per group. Significance was determined using a two-tailed Student’s *t* test, ^#^*P* = 0.004. **h** Schematic of breeding the Tmx-inducible *Pax7*^*Cre-ERT2*^ mice with *Rosa26-DTA* targeted mice. These lines were crossed onto the *δ-sarcoglycan*–null (*Sgcd*^–/–^) background. **i** Schematic representation of the Tmx treatment regimen. Two-month-old mice received Tmx injections for five consecutive days and were subsequently placed on Tmx chow until harvest at 4 months of age. The key shows the 3 genotypes examined by color for the remaining panels. **j** Quantification of Pax7^+^ satellite cells in muscle sections from the Quad of mice with the indicated genotypes. *n* = 4 mice per group. A 1-way ANOVA with Tukey’s multiple comparisons test was used to determine significance, ***P < 0.001 vs Wt, ^#^*P* < 0.001 vs *Sgcd*^*−/−*^. The 4 month Wt and *Sgcd*^*−/−*^ Pax7 positive counts are also shown in Fig. 2l. **k** Quantification of the number of Myh3 positive fibers in Quad muscle sections of mice with the indicated genotypes. *n* = 4 for both groups. Significance was determined using a two-tailed Student’s *t* test, ^#^*P* = 0.01. The 4 month *Sgcd*^*−/−*^ Myh3 positive fiber counts are also shown in Fig. 2n. **l** Representative Quad muscle sections immunostained for Myh3 (red) and laminin (green) in 4-month-old mice of the indicated genotypes. Dapi stained nuclei are in blue. Scale bar = 100 μm. **m** Muscle weights (MW) normalized to tibia length (TL) from mice of the indicated genotypes at 4 months of age. *n* = 9 Wt mice; *n* = 4 *Sgcd*^*−/−*^ and *Sgcd*^*−/−*^; *R26-DTA*^*Pax7Cre-ER*^ mice. A 1-way ANOVA with Tukey’s multiple comparisons test was used to determine significance, **P < 0.01 vs Wt, ***P < 0.001 vs Wt, ^##^*P* < 0.001 vs *Sgcd*^*−/−*^. **n** Representative H&E-stained sections of the Quad muscle from 4-month-old mice of the indicated genotypes. Scale bar = 100 μm. **o** Quantification of IgM positive fibers in muscle sections from mice of the indicated genotypes. *n* = 5 *Sgcd*^*−/−*^ mice and *n* = 4 *Sgcd*^*−/−*^; *R26-DTA*^*Pax7Cre-ER*^ mice. Significance was determined using a two-tailed Student’s *t* test, ^#^*P* = 0.01. Data represent mean ± SEM for all graphs.

To determine whether the loss of satellite cells could similarly reduce muscle pathology in a second dystrophic mouse model, we crossed the *Mapk3*^*−/−*^; *Mapk1*^*f/f-Pax7Cre-ER*^ alleles onto the DMD *mdx*^4Cv^ background^15^. We treated *mdx; Mapk3*^*−/−*^*; Mapk1*^*f/f-Pax7Cre-ER*^ and *mdx* control mice with tamoxifen starting at 2 weeks of age through 8 weeks, which again results in a dramatic depletion of satellite cells versus control mice without the *Pax7*^*Cre-ER*^ allele (Supplementary Fig. 4a, b). Satellite cell depleted *Mapk3*^*−/−*^*; Mapk1*^*f/f-Pax7Cre-ER*^ mice again showed an absence of Myh3 positive myofibers and myofibers with centrally located nuclei, as well as noticeably improved histopathology including myofiber hypertrophy (Supplementary Fig. 4c–f). This same profile of disease protection was also observed in young adult *mdx* mice with satellite cell depletion between 2 and 4 months of age (Fig. 3a). At this later time point, satellite cells were again completely eliminated and Myh3 positive de novo myofibers were absent in four-month-old *mdx; Mapk3*^*−/−*^*; Mapk1*^*f/f-Pax7Cre-ER*^ mice relative to controls (Fig. 3b–d). Muscle weights of *mdx; Mapk3*^*−/−*^*; Mapk1*^*f/f-Pax7Cre-ER*^ mice were like Wt controls but significantly smaller than the pseudo-hypertrophied muscles observed in *mdx* controls (Fig.3e). However, myofibers from *mdx; Mapk3*^*−/−*^*; Mapk1*^*f/f-Pax7Cre-ER*^ mice were larger in size relative to those from Wt and mdx controls (Fig. 3f). Finally, as we described in *Sgcd*^*−/−*^ mice lacking satellite cells, a significant reduction in IgM positive myofibers was observed in histological sections of *mdx* mice without satellite cells compared to muscle from *mdx* mice with satellite cells (Fig. 3g). However, a limitation in our analysis of the *mdx* model is the lack of assessment of muscle function and CK serum levels.

### Diphtheria toxin-mediated satellite cell ablation mitigates MD in Sgcd^−/−^ mice

We generated an Erk1/2 independent model for satellite cell ablation in the *Sgcd*^*−/−*^ background that depends on expression of the diphtheria toxin from the *Rosa26* locus (Fig. 3h)^16^ However, for this approach we used a different *Pax7*^*Cre-ER*^ mouse that has an IRES-Cre cassette downstream of the endogenous stop codon that did not cause lethality when crossed with the *Rosa26-DTA* allele^17^. Two-month-old mice were given tamoxifen for two months to induce depletion of satellite cells in early adulthood (Fig. 3i). Relative to controls, *Sgcd*^*−/−*^; *R26-DTA*^*Pax7Cre-ER*^ mice had a significant reduction in satellite cells as assessed by immunostaining for Pax7 in histological sections (Fig. 3j), which also significantly reduced the number Myh3 positive regenerating myofibers (Fig. 3k, l), restored muscle weights to normal (Fig. 3m), improved tissue histopathology (Fig. 3n) and improved membrane stability as measured with IgM accessibility (Fig. 3o). The results obtained using *Sgcd*^*−/−*^; *R26-DTA*^*Pax7Cre-ER*^ mice are consistent with the results observed with *Mapk1/3* deletion, again showing that dystrophic myofibers lacking satellite cells show reduced MD pathology.

### Satellite cell activity in MD destabilizes the sarcolemma through a MyoD-dependent myogenic fetal gene program

**Fig. 4:**
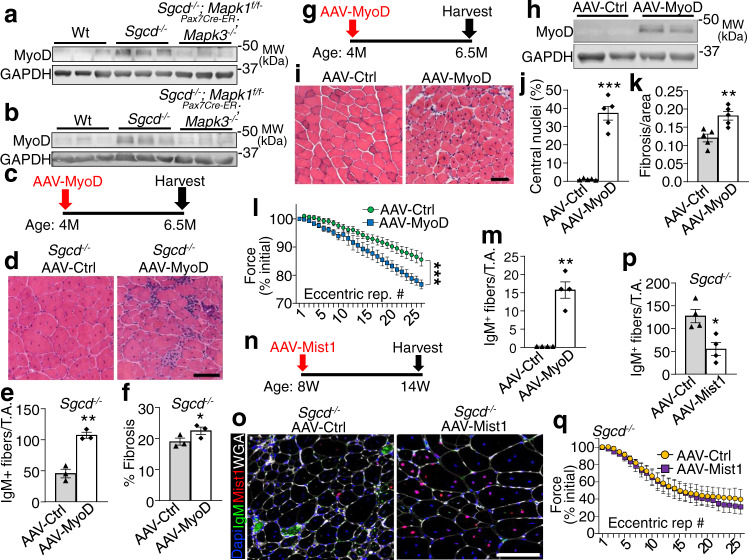
Ectopic MyoD expression destabilizes the sarcolemma in vivo. Western blotting for MyoD protein expression using Quad lysate from 2- or 4-month-old mice of indicated genotypes treated with Tmx starting at 2 (**a**) or 8 (**b**) weeks of age. GAPDH is shown as loading control. Results from three different mice are shown. **c** Schematic for AAV-MyoD or AAV-control injection into *Sgcd*^*−/−*^ mice at 4 months of age, harvested by 6.5 months. **d** H&E-stained TA muscle histological sections from *Sgcd*^*−/−*^ mice injected with the indicated recombinant virus. Scale bar = 100 μm. **e** IgM positive fibers quantified in TA muscle histological sections with prior AAV-MyoD or AAV-control infection in *Sgcd*^*−/−*^ mice. *n* = 3 mice per group, **P < 0.01 vs AAV-Ctrl, two-tailed Student’s *t* test. **f** Picrosirius red-stained histological section of the TA muscle for quantitation of fibrosis in *Sgcd*^*−/−*^ mice with prior AAV-MyoD or AAV-control infection. *n* = 3 mice per group, *P < 0.05 vs AAV-Ctrl, two-tailed Student’s *t* test. **g** Schematic of recombinant AAV treatment in Wt mice. **h** Western blot for MyoD from TA muscle of Wt mice 2.5 months after AAV injection. GAPDH was run as a loading control. Results from two different mice are shown. **i** H&E-stained TA muscle histological sections from Wt mice injected with the indicated recombinant virus. Scale bar = 100 μm. **j** Quantitation of myofiber number with central nucleation in H&E-stained histological sections from the TA muscle with prior AAV-MyoD or AAV-control infection in Wt mice. *n* = 5 mice per group, ***P < 0.001 vs AAV-Ctrl, two-tailed Student’s *t* test. **k** Quantitation of fibrosis in picrosirius red-stained histological sections from the TA muscle in Wt mice with prior AAV-MyoD or AAV-control infection. *n* = 5 mice per group, **P = < 0.01 vs AAV-Ctrl, two-tailed Student’s *t* test. **l** Percent change in force transduction for TA muscles subjected to continuous repetitive eccentric contractions in Wt mice injected previously with AAV-MyoD or AAV-Ctrl. The *x*-axis shows number of progressive repetitive (rep) contractions. A two-way repeated measures ANOVA was used to determine significance, *n* = 12 mice for AAV-Ctrl, *n* = 11 mice for AAV-MyoD, ***P < 0.001 for interaction. **m** IgM positive fibers quantified in TA muscle histological sections with prior AAV-MyoD or AAV-control injection in Wt mice. *n* = 4 mice per group, **P < 0.001 vs AAV-Ctrl, two-tailed Student’s *t* test. **n** Schematic of recombinant AAV-Mist1 vs AAV-Ctrl TA muscle injection in *Sgcd*^*−/−*^ mice at the indicated times. **o** Immunohistochemistry from the TA muscle of *Sgcd*^−/−^ mice injected prior with AAV-Mist1 or AAV-Ctrl, stained for IgM (green), Mist1 (red), Dapi for nuclei (blue) or myofiber outlines with wheatgerm agglutinin (white). Scale bar = 100 μm. **p** IgM positive fibers quantified in TA muscle histological sections of *Sgcd*^*−/−*^ mice with prior AAV-Mist1 or AAV-control injection. mice. *n* = 4 mice per group, *P < 0.05 vs AAV-Ctrl, two-tailed Student’s *t* test. **q** Percent change in force transduction in the hindlimb subjected to continuous repetitive eccentric contractions in *Sgcd*^*−/−*^ mice injected previously with AAV-Mist1 or AAV-Ctrl. The *x*-axis shows number of progressive repetitive (rep) contractions. *n* = 7 mice for AAV-Ctrl, *n* = 8 mice for AAV-Mist1. Data represent mean ± SEM for all graphs.

Here we examined the mechanism whereby satellite cell activity further destabilizes the myofiber sarcolemma in conjunction with an existing genetic mutation associated with MD. We observed increased MyoD expression within dystrophic muscles at the level of the myofibers from *Sgcd*^*−/−*^ mice (Supplementary Fig 5a), which was abrogated by previous deletion of satellite cells (Fig. 4a, b). MyoD is known to regulate expression of fetal like genes during myoblast to myofiber differentiation^18^ including myocyte derived myomaker that regulates sarcolemmal stability to promote myoblast fusion to myofibers^19^. To directly test the hypothesis that the MyoD-dependent gene program drives pathogenic excess and sarcolemma destabilization, we generated a recombinant adeno-associated virus serotype-9 driving MyoD (AAV-MyoD vs. AAV-control empty vector) that was directly injected into the right vs left tibialis anterior (TA) muscle of 4-month-old dystrophic *Sgcd*^*−/−*^ mice, followed by analysis 2.5 months later (Fig. 4c). Sustained MyoD expression in the TA of *Sgcd*^*−/−*^ mice worsened muscle histopathology, led to greater membrane destabilization with IgM positivity, and greater fibrosis (Fig. 4d–f). Remarkably, even Wt mice subjected to AAV-MyoD protein induction from 4 to 6.5 months (Fig. 4g, h) exhibited MD related pathology (i.e., central nucleation, sarcolemmal permeability to IgM, fibrosis, and increased susceptibility to eccentric contraction injury) compared with control AAV infection (Fig. 4i–m). In a reciprocal approach, we used AAV9-Mist1 inhibitory protein overexpression to suppress endogenous MyoD in the TA of *Sgcd*^*−/−*^ mice between 8 and 14 weeks of age (Fig. 4n). As with AAV-MyoD, we similarly observed efficient expression of Mist1 throughout the myofibers of the infected TA muscle (Fig. 4o). While Mist1 overexpression yielded a significant reduction in IgM myofiber positivity (Fig. 4p), protection from eccentric induced force loss was not observed suggesting that the Mist1 inhibitory approach had only a partial effect in reducing the pathology associated with MyoD activity (Fig. 4q).

To investigate the mechanism whereby MyoD expression induces or worsens MD pathology, we generated a C2C12 myotube cell culture model with doxycycline-inducible MyoD expression from a recombinant lentivirus, followed by mRNA expression profiling (with or without MyoD overexpression (+Dox)) (Supplementary Fig 5b). The data revealed dysregulation of genes belonging to select functional groups such as myotube differentiation, muscle organ development, and the actin cytoskeleton (Supplementary Fig 5b). We also observed significant changes in expression of membrane associated genes with MyoD overexpression that could have a role in negatively impacting the sarcolemma in MD, such as Fabp3^20^, Dysf^21^, Myot^22^, Mymk^19^, Xirp1^23^, Cav3^24^, Trim72^25^, Megf10^26^ (Supplementary Fig 5b). Indeed, direct analysis of mRNA expression in the TA muscle from either AAV-MyoD or AAV-Mist1 injected mice showed significant regulation of the membrane fusion effectors, myomaker and myomerger (Supplementary Fig 5c, d), which is especially relevant given that specific deletion of myomaker in myofibers was recently shown to reduce muscular dystrophy pathological features in the mouse^19^ (see discussion below).

## Discussion

In adult skeletal muscle, resident satellite cells are largely quiescent until acute muscle fiber damage or death. This elicits satellite cell activation and directs a MyoD-dependent reactivation of the fetal gene program in muscle fibers, promoting myoblast fusion events necessary for repair or regeneration of the muscle. However, it is unlikely that satellite cells evolved to deal with rare monogenic diseases with chronic tissue wasting and inherent sarcolemma fragility like the MDs. While satellite cell-mediated regeneration is commonly considered essential and beneficial in MD pathogenesis, our current findings challenge this dogmatic assumption and suggest that satellite cells are not exclusively protective.

While *Sgcd*^*−/−*^ mice depleted of satellite cells at 2 months of age appear unaffected up to 7 months of age, depletion beginning at 2 weeks of age during neonatal development results in substantially less muscle mass in these *Sgcd*^*−/−*^ mice, and they eventually show decline in later months from muscle loss. However, a recent study suggests that mature myofibers can repair after exercise without the need for satellite cells, through a process intrinsic to the myofiber and an ability to move existing differentiated myonuclei to the site of injury^27^. Furthermore, deletion of satellite cells in adult Wt mice did not lead to sarcopenia over 20 months time, suggesting that satellite cells are not needed for long-term muscle maintenance in a caged environment^28^. Hence, the seemingly “logical” concept that muscle cannot survive long-term without satellite cells is complex such that Wt mice or uninjured mice appear stable without these cells. However, early neonatal depletion of satellite cells at 2 weeks of age in conjunction with an underlying chronic dystrophic disease state leads to reduced muscle and mortality with aging.

Earlier work did suggest that perhaps satellite cells were not strictly beneficial for muscle survival in MD^2^. It was shown that irradiation of one limb to inactivate satellite cells in a mouse model of MD produced improved muscle histopathology compared with the non-irradiated leg. Since this time other suggestive evidence has emerged in the literature whereby satellite cells could be detrimental in MD. For example, deletion of the membrane fusion factor myomaker from myofibers, which is induced by MyoD, protected dystrophic mice from damage at the level of the sarcolemma^19^.

Here we demonstrated that forced MyoD re-expression in otherwise Wt muscle leads to dystrophic features and reduced membrane stability, while MyoD overexpression exacerbates MD and inhibition of MyoD activity reduced IgM myofiber positivity in the *Sgcd*^*−/−*^ background. Interestingly, *Myod1*-null mice were reported to have worse dystrophic disease in the *mdx* background, which would be the opposite of our predicted mechanism of action. However, *Myod1*-null mice had a greater population of satellite cells and myoblasts^29,30^. In fact, *mdx* mice lacking the *Myod1* gene have significantly more satellite cells compared to *mdx* controls and based on our results this could underlie greater satellite cell activity and disease, but in this case presumably based on a Myf5-dependent fetal gene program induction^31^.

By depleting satellite cells and their induction of the MyoD-dependent fetal gene program in myofibers using either of 2 separate genetic approaches in 2 genetic models of MD, we demonstrate an overwhelming effect on sarcolemmal membrane stability. The MDs as a group are most typically associated with genetic mutations in genes that underlie some aspect of sarcolemmal membrane support, repair, or the activity of select channels and signaling components within the membrane^32^. Our results suggest that these underlying genetic defects that comprise the MDs only represent the first “hit” in a “2-hit” model, with the second hit being that of additional destabilization associated with induction of the myogenic fetal gene program due to new satellite cell activity and myoblast fusion. This membrane instability induced by MyoD is likely necessary to support new and continual myonuclei accretion as part of the attempted repair process.

While a therapeutic approach aimed at depleting satellite cells in MD patients is ill-advised at this point in time, it does not mean that a valuable future treatment is beyond imagination, given the appreciated basic science shown here. Indeed, satellite cell-mediated induction of the fetal gene program and associated sarcolemmal instability in myofibers during MD could represent more specific therapeutic targets of value, especially as we define the most critical molecular effectors of these disease processes (such as MyoD and membrane altering genes).

## Methods

### Animal models

This study was performed entirely in mice, using transgenic or gene-targeted models either commercially available or generated as described below. No human subjects or human material was used. All experiments involving mice were approved by the Institutional Animal Care and Use Committee (IACUC) at Cincinnati Children’s Hospital under protocol IACUC2018-0047. All procedures were performed in compliance with institutional and governmental regulations under PHS Animal Welfare Assurance number D16-00068 (A3108-01). Mice did not undergo randomization because they were genetically identical and many groups were from the same litters, as well as matched for age and sex ratio. Both male and female mice were used in an equal ratio, and no sex-specific differences were observed. However, mdx^4Cv^ mice required the use of males given the location of the dystrophin gene on the X chromosome. Mice were housed in a barrier facility with 14 h daylight and 10 h darkness, with ad libitum access to water and food (Teklad Global 19% Protein Extruded Rodent Diet).

Mice containing a genetic insertion of a tamoxifen-inducible Cre recombinase–estrogen receptor fusion protein, CreER^T2^ cDNA within the Pax7 *locus* (Jax stock No: 012476)^9^ were crossbred with *Mapk3*^*−/−*^ (Erk1, Jax stock No: 019113)^7^ and *Mapk1*^*f/f*^ (Erk2, Jax stock No: 019112) mice^8^. *Mapk3*^*−/−*^; *Mapk1*^*f/f-Pax7Cre-ER*^ were crossed with *Rosa26* loxP site-dependent reporter mice (*R26eGFP*, Jax Stock No: 012429) to visualize satellite cells as well as onto the *Sgcd*^*−/−*^ (Jax stock No: 004582)^13^ or the *mdx*^4Cv^ backgrounds (Jax stock No: 002378)^15^ to study the effects of satellite cell depletion in dystrophic mouse models. Other mouse lines used in the study were: *Rosa26-DTA* (R26-DTA, Jax stock No: 006331)^33^, and *Pax7*^*CreERT2*^ (Jax stock No: 017763)^17^ mice. All mice were backcrossed into the *C57Bl*/6 genetic background for at least 4 generations, including the *mdx*^4Cv^ line.

### Animal procedures

Tamoxifen was administered to all experimental and control mice via intraperitoneal injections (75 mg/kg) using pharmaceutical-grade tamoxifen (Sigma) dissolved in corn oil (Sigma). Subsequently, in experiments requiring longer term tamoxifen regimens, the mice were fed a diet containing 400 mg/kg tamoxifen citrate (Envigo, TD.55125) for the indicated period of time. Muscle injury was induced by injecting the TA muscle with 50 μl of cardiotoxin (10 µmol/L, Sigma).

### Western blotting

Muscles were homogenized and lysates prepared as previously described^34^. Proteins were resolved by SDS-PAGE, transferred to Immobilon-FL membranes (Millipore) and incubated overnight with antibodies against, phosphorylated Erk1/2 (1:1000, Cell Signaling Technology), Erk1/2 (1:2000, Cell Signaling Technology), MyoD (1:200, BD Bioscience) and GAPDH (1:500 000, Fitzgerald), Membranes were then incubated with IRDye secondary antibodies (1:6000, LI-COR Biosciences) and visualized using an Odyssey CLx imaging system (LI-COR Biosciences).

### Immunofluorescence

Histological sections (8 µm) were collected from frozen skeletal muscles using a cryostat and fixed in 4% paraformaldehyde (PFA) for 10 min. Sections were subsequently washed in 1X phosphate buffer saline (PBS) and heated in citrate buffer (pH 6.0) for 20 min and maintained in blocking buffer for 30 min (10% goat serum and 0.4% triton X diluted in 1X PBS). Slides were stained overnight at 4 °C with antibodies diluted in staining solution (1% bovine serum albumin, 0.04% triton X diluted in 1X PBS). Satellite cells were visualized by staining for Pax7 (1:10, Developmental Studies Hybridoma Bank (DSHB)) and regenerating myofibers were identified by staining for Myh3 (F1.652, 1:10, DSHB). Myofibers present in a muscle section in Fig. 1d were stained with an anti-myosin antibody (MF-20, 1:40, DSHB) while anti-laminin antibody (1:200, Sigma) was used to visualize the outline of all myofibers present in a given muscle section. Mist1 antibody (1:1000, Cell Signaling Technology) was also used in Fig. 4o, while MyoD antibody (5.8A, 1:100, Santa Cruz Biotechnologies) was used in Supplementary Fig 5a. Nuclei were visualized using Dapi (Invitrogen). IgM primary antibody conjugated to FITC (1:300, Sigma) was used to highlight myofibers with compromised membrane integrity. TA muscles analyzed for eGFP reporter activity were fixed in 4% PFA for 4 h at 4 °C and then washed in 1X PBS and equilibrated overnight in 30% sucrose diluted in 1X PBS. Sections were stained for eGFP (Novus).

Primary antibodies were visualized using Alexa-568 and 488 goat anti-rabbit IgG (Invitrogen), Alexa-488 goat anti-mouse IgG2b (Invitrogen) and Alexa-568 anti-mouse IgG1 (Invitrogen) secondary antibodies diluted 1:500 in staining solution. Immunofluorescence images were captured using a Nikon Eclipse Ti microscope or a Nikon A1R confocal microscope using NIS Elements AR 4.13 software. Data relating to satellite cell counts were generated by counting Pax7 positive cells relative to each myofiber present in two 10X images. Regenerating myofibers were assessed by counting Myh3 positive myofibers from an entire muscle section at mid-belly for a given animal. IgM positive myofibers were counted from entire muscle sections taken at the mid-belly of the muscle and normalized to muscle section area. All analyses were performed in a blinded fashion whereby the experimenter was only made aware of the genotypes following proof of quantification.

### Pathological indices

Histological cross-sections (8 μm) were collected from skeletal muscles using a cryostat and stained for H&E or Picrosirius Red to assess myofibers with centrally located nuclei and fibrosis, respectively. The number of myofibers with centrally located nuclei was quantified from two 10X micrographs taken from histological sections of the quadriceps at the mid-belly. Fibrosis was quantified from two 10X pictures taken from histological sections of the quadriceps. Quantification of fibrotic area was calculated using the ImageJ analysis software (National Institutes of Health). The minimal feret’s diameter was determined from laminin stained muscle sections using ImageJ. All analyses were performed in a blinded fashion whereby the experimenter was only made aware of the genotypes following proof of quantification.

### Affymetrix microarray and bioinformatics

Total RNA was extracted from differentiated (72 h) C2C12 cells (mouse myoblast cell line from ATCC, product CRL-1772) using the RNeasy Kit (Qiagen) according to the manufacturer’s protocol. RNA quality and integrity were assessed using the Agilent 2100 Bioanalyzer (Gene Expression Core (GEC) Facility (Cincinnati Children’s Hospital)). Microarray analysis of these RNA samples was carried out at the GEC facility using Affymetrix Clariom S arrays. Bioinformatics analysis of resultant CHP files to determine differential gene expression profiles was carried out using the Transcriptome Analysis Console (Applied Biosystems; ver. 4.0.0.25), the Clariom_S_Mouse TAC Configuration file (ver. 4), and the iPathwayGuide (Advaita Bioinformatics). Heat maps were generated from gene-signals utilizing the Signal Space Transformation Robust Multi-Array Average normalization method (sst-rma-gene-signals) for each gene of interest using Heatmapper software.

### Cell culture

C2C12 cells and HEK293 cells (human embryonic kidney cells, ATCC product #CRL-1573) were purchased from American Type Culture Collection. Cells were cultured in DMEM (Thermo Fisher Scientific) supplemented with penicillin-streptomycin (Thermo Fisher Scientific), sodium pyruvate (Sigma) and either 10% bovine growth serum (Thermo Fisher Scientific) for C2C12 or 10% fetal bovine serum (VWR) for HEK293 cells. Cultures were maintained in a humidified incubator at 37 °C and 5% CO_2_ and split every 48–72 h upon trypsinization using 0.05% trypsin-EDTA (Thermo Fisher Scientific). For differentiation, C2C12 cells were cultured in DMEM media containing the same antibiotics and sodium pyruvate, but with 2% horse serum (Life Technologies), which was replaced every 24 h.

### Lentivirus generation and transduction

The MyoD cDNA (Addgene plasmid # 14710) was subcloned using the In-Fusion system (Clontech, # 638910) into a pLVX-TetON transfer vector (Clonetech) that was modified to be blasticidin resistant. To generate lentiviruses, 293T cells (human embryonic kidney cells, ATCC product # CRL-3216) grown in 10 cm plates (3.8 × 10^6^ cells/plate) containing culture media described above. Immediately before transfection, media was replaced with 9.5 mL of fresh culture media, that was antibiotic-free and cells transfected with Fugene6 (Promega) using 10 μg of either pLVX-TetON-MyoD or pLVX-TetON-empty vector, 5 μg of psPAX2 (Addgene), and 5 µg of pCMV-VSV-G (Addgene) mixed with Fugene6 in a ratio of 3 µL:1 µg DNA. Virus supernatants were harvested 48 and 72 h post-transfection, filtered using a 0.45 µm sterile, surfactant-free filter and virus concentrated by ultracentrifugation at 100,000 × *g* using a SW 32Ti swinging-bucket rotor at 4 °C for 2 h. Viral pellets were resuspended in 200 µL of complete medium and maintained on ice for immediate use.

For transduction, 50,000 C2C12 cells were plated per well in a 6-well format and cultured for ~20 h prior to transduction. Media was replenished (1.8 mL/well) immediately prior to transduction and supplemented with polybrene at 6 µg/mL. Freshly concentrated lentiviral preps (200 µL) were added to the media and gently mixed using a pipetman. Cells were then subjected to “spinfection” at 652 × *g* for 1 h at 22 °C, and transduction allowed to proceed for an additional 24 h under normal culture conditions. Media was replaced with fresh culture medium containing 10 μg/mL blasticidin (Thermo Fisher Scientific) and cells subsequently cultured in blasticidin containing media to maintain selection pressure. After 18 h, media was switched to 2% horse serum containing differentiation medium with or without 1 µg/mL doxycycline for a total of 72 h to induce expression of MyoD. C2C12 cells were then harvested and RNA was collected for gene expression analysis as described above.

### AAV9 viral production and injection into mice (TA muscle)

The cDNA for MyoD (Addgene, plasmid # 14710) and Mist1 (accession # BC094061) were subcloned individually into the pAAV-MCS Expression Vector (Cell Biolabs Inc., # VPK-410) with the In-Fusion HD Cloning Plus Kit (Clontech, # 638910). The insertion of the cDNAs was confirmed by sequencing and the plasmids were amplified overnight in DH5α *Escherichia coli* (ThermoFisher, # 18265017), purified using the PureYield Plasmid Maxiprep System (Promega, #A2392) and were sent to our in-house viral core for packaging and large scale purification. A third virus was made using pAAV-MCS and served as an empty vector control (AAV-Ctrl). Adult C57BL/6 and *Sgcd*^*−/−*^ mice were administered either AAV9-MyoD, AAV-Mist1 or AAV-Ctrl via an intramuscular injection into the TA muscle at a concentration of 1 × 10^12^ viral particles in 40 µl of sterile PBS under inhaled isoflurane anesthesia to effect. Prior to injection, mice were treated with a hair removal product (Nair) at the site of injection.

### Reverse-transcriptase PCR

RNA was extracted from flash frozen TA muscles using the RNeasy kit (Qiagen) according to the manufacturer’s protocol. Total RNA was reverse transcribed using random oligo-dT primers and Superscript Reverse Transcriptase (Thermo Fisher Scientific). qPCR was performed using SsoAdvanced SYBR Green (Bio-Rad, 6090), and the cycle threshold values were normalized to the values obtained from RPL7. All experiments were performed in duplicate. The following primer sets were used to identify transcripts: MyoD, 5′- TGGCATGATGGATTACAGCG, 5′- CCACTATGCTGGACAGGCAGT; Mist1, 5′- AGGCAGCGGATGCATAAA, 5′- GCGGTCAGCGACTTGATATAG; Mymk, 5′- TTCCTCCCGACAGTGAGCAT, 5′- GCACAGCACAGACAAACCAG; Mymx, 5′- GTGTTGATTGACCCTTCCTCTC, 5′- CTGACCTTGATACCCGCATTT; Cav3, 5′- GGATCTGGAAGCTCGGATCAT, 5′- TCCGCAATCACGTCTTCAAAAT. Rpl7 5’- GAAGCTCATCTATGAGAAGGC; 5’- AAGACGAAGGAGCTGCAGAAC. Data are fold change relative to AAV-Ctrl injected samples.

### Serum creatine kinase measurement

Blood samples from mice were collected and plasma was isolated by centrifugation at 5000 rpm for 15 min at 4 °C. Serum creatine kinase measurement was performed on a Roche cobas c 311 clinical chemistry analyzer (Roche).

### Treadmill running

Mice were subjected to forced down-hill treadmill running using a ramping speed protocol and failure events were noted a shock pad engagement, as previously described^14^. Mice were run until the entire protocol was completed, which was typically 10 min.

### Hindlimb force frequency analysis with repetitive eccentric contractions of hindlimb

Muscle performance was measured in vivo with a 305C muscle lever system (Aurora Scientific Inc., Aurora, CAN), as previously described^35,36^. Animals were anesthetized via inhalation (~3% isoflurane, SomnoSuite, Kent Scientific), and placed in a thermostatically controlled table with anesthesia maintained via nose-cone (~2% isoflurane). The knee was first isolated using a pin through the tibial head and the foot firmly fixed to a footplate on the motor shaft. For assessment of plantarflexor function, contractions were elicited by percutaneous electrical stimulation of the tibial nerve. For assessment of dorsiflexor function, contractions were elicited by percutaneous electrical stimulation of the peroneal nerve. Optimal isometric twitch torque determined by increasing the current with a minimum of 30 s between each contraction to avoid fatigue. A series of stimulations were performed at increasing frequency of stimulation (0.2 ms pulse, 500 ms train duration): 1, 20, 40, 60, 80, 100 and 150 Hz. Following assessment of isometric torque, susceptibility to injury was assayed with 25 eccentric contractions as previously described^35,36^ at maximal isometric torque (150 ms duration, 0.2-ms pulse train at 150 Hz). Eccentric contractions were achieved by translating the footplate 40° backward at a velocity of 800°/s after the first 100 ms of the isometric contraction. The decrease in the peak isometric force before the eccentric phase was taken as an indication of muscle damage.

### Statistical analysis and experimental rigor

A one-way analysis of variance was used to determine if there was a significant difference in experiments with more than 2 groups; a Tukey’s post hoc test was performed to compare individual groups (Prism Software). Significant differences between 2 groups were determined using a two-tailed unpaired Student’s *t* test or a paired *t* test depending on if the groups were related (same mouse), or unrelated by 2 different examinations (Stats Plus Software). Significance was set at *P* < 0.05 for all experiments. Significant differences in muscle function or eccentric injury was determined using two-way repeated measures ANOVA with a Holm-Sidak post-hoc when appropriate. All results are presented as mean and the error bar represents the standard error of the mean.

## Supplementary information

Supplementary Information

## Data Availability

All raw data generated or analyzed in this study are included in the study. Original source data used to generate graphs in each of the figures and supplementary figures are provided as Microsoft Excel data sheet files. Expression microarray data were deposited in the Gene Expression Omnibus (GEO) as accession number GSE168703 (https://www.ncbi.nlm.nih.gov/geo/). Source data are provided with this paper.

## Source data

Source Data

## References

[1] Yin H, Price F, Rudnicki MA (2013). Satellite cells and the muscle stem cell niche. Physiol. Rev..

[2] Granata AL (1998). Gamma irradiation can reduce muscle damage in mdx dystrophic mice. Acta Neuropathol..

[3] Rossi G (2016). Nfix regulates temporal progression of muscle regeneration through modulation of myostatin expression. Cell Rep..

[4] Rossi G (2017). Silencing Nfix rescues muscular dystrophy by delaying muscle regeneration. Nat. Commun..

[5] Jones NC, Fedorov YV, Rosenthal RS, Olwin BB (2001). ERK1/2 is required for myoblast proliferation but is dispensable for muscle gene expression and cell fusion. J. Cell Physiol..

[6] Machado, L. et al. Tissue damage induces a conserved stress response that initiates quiescent muscle stem cell activation. *Cell Stem Cell*, 10.1016/j.stem.2021.01.017 (2021). 10.1016/j.stem.2021.01.017 33609440

[7] Pages G (1999). Defective thymocyte maturation in p44 MAP kinase (Erk 1) knockout mice. Science.

[8] Samuels IS (2008). Deletion of ERK2 mitogen-activated protein kinase identifies its key roles in cortical neurogenesis and cognitive function. J. Neurosci..

[9] Lepper C, Conway SJ, Fan CM (2009). Adult satellite cells and embryonic muscle progenitors have distinct genetic requirements. Nature.

[10] Yamamoto M (2009). A multifunctional reporter mouse line for Cre- and FLP-dependent lineage analysis. Genesis.

[11] Pawlikowski B, Pulliam C, Betta ND, Kardon G, Olwin BB (2015). Pervasive satellite cell contribution to uninjured adult muscle fibers. Skelet. Muscle.

[12] White RB, Bierinx AS, Gnocchi VF, Zammit PS (2010). Dynamics of muscle fibre growth during postnatal mouse development. BMC Dev. Biol..

[13] Hack AA (2000). Differential requirement for individual sarcoglycans and dystrophin in the assembly and function of the dystrophin-glycoprotein complex. J. Cell Sci..

[14] Goonasekera SA (2011). Mitigation of muscular dystrophy in mice by SERCA overexpression in skeletal muscle. J. Clin. Invest.

[15] Chapman VM, Miller DR, Armstrong D, Caskey CT (1989). Recovery of induced mutations for X chromosome-linked muscular dystrophy in mice. Proc. Natl Acad. Sci. USA.

[16] Lepper C, Partridge TA, Fan CM (2011). An absolute requirement for Pax7-positive satellite cells in acute injury-induced skeletal muscle regeneration. Development.

[17] Murphy MM, Lawson JA, Mathew SJ, Hutcheson DA, Kardon G (2011). Satellite cells, connective tissue fibroblasts and their interactions are crucial for muscle regeneration. Development.

[18] Blais A (2005). An initial blueprint for myogenic differentiation. Genes Dev..

[19] Petrany, M. J., Song, T., Sadayappan, S. & Millay, D. P. Myocyte-derived Myomaker expression is required for regenerative fusion but exacerbates membrane instability in dystrophic myofibers. *JCI Insight* **5**, 10.1172/jci.insight.136095 (2020). 10.1172/jci.insight.136095 PMC7253022 32310830

[20] Lee SM (2020). FABP3-mediated membrane lipid saturation alters fluidity and induces ER stress in skeletal muscle with aging. Nat. Commun..

[21] Glover LE (2010). Dysferlin overexpression in skeletal muscle produces a progressive myopathy. Ann. Neurol..

[22] Garvey SM, Liu Y, Miller SE, Hauser MA (2008). Myotilin overexpression enhances myopathology in the LGMD1A mouse model. Muscle Nerve.

[23] Otten C (2012). Xirp proteins mark injured skeletal muscle in zebrafish. PLoS One.

[24] Cai C (2009). Membrane repair defects in muscular dystrophy are linked to altered interaction between MG53, caveolin-3, and dysferlin. J. Biol. Chem..

[25] Cai C (2009). MG53 regulates membrane budding and exocytosis in muscle cells. J. Biol. Chem..

[26] Draper I (2019). The impact of Megf10/Drpr gain-of-function on muscle development in Drosophila. FEBS Lett..

[27] Roman W (2021). Muscle repair after physiological damage relies on nuclear migration for cellular reconstruction. Science.

[28] Fry CS (2015). Inducible depletion of satellite cells in adult, sedentary mice impairs muscle regenerative capacity without affecting sarcopenia. Nat. Med..

[29] Megeney LA, Kablar B, Garrett K, Anderson JE, Rudnicki MA (1996). MyoD is required for myogenic stem cell function in adult skeletal muscle. Genes Dev..

[30] Macharia R, Otto A, Valasek P, Patel K (2010). Neuromuscular junction morphology, fiber-type proportions, and satellite-cell proliferation rates are altered in MyoD(−/−) mice. Muscle Nerve.

[31] Rudnicki MA, Braun T, Hinuma S, Jaenisch R (1992). Inactivation of MyoD in mice leads to up-regulation of the myogenic HLH gene Myf-5 and results in apparently normal muscle development. Cell.

[32] Wallace GQ, McNally EM (2009). Mechanisms of muscle degeneration, regeneration, and repair in the muscular dystrophies. Annu Rev. Physiol..

[33] Ivanova A (2005). In vivo genetic ablation by Cre-mediated expression of diphtheria toxin fragment A. Genesis.

[34] Wissing ER (2014). P38alpha MAPK underlies muscular dystrophy and myofiber death through a Bax-dependent mechanism. Hum. Mol. Genet.

[35] Call JA, Lowe DA (2016). Eccentric contraction-induced muscle injury: reproducible, quantitative, physiological models to impair skeletal muscle’s capacity to generate force. Methods Mol. Biol..

[36] Khairallah RJ (2012). Microtubules underlie dysfunction in duchenne muscular dystrophy. Sci. Signal.

