# Supplementary material for: Depletion of skeletal muscle satellite cells attenuates pathology in muscular dystrophy
Source: Nat Commun. 2022 May 26;13:2940. doi: 10.1038/s41467-022-30619-7 (PMC9135721; doi:10.1038/s41467-022-30619-7)
Supplement: Supplementary file 1 — Supplementary Information [file 41467_2022_30619_MOESM1_ESM.pdf]

Supplementary Information

Deletion of skeletal muscle satellite cells attenuates pathology in muscular dystrophy

Justin G. Boyer[1,2], Jiuzhou Huo[1], Sarah Han[1], Julian R. Havens[1,2], Vikram Prasad[1], Brian L. Lin[3], David A Kass[3], Taejeong Song[4], Sakthivel Sadayappan[4], Ramzi J. Khairallah[5], Christopher W. Ward[6], Jeffery D. Molkentin[1,2*]

[1]Department of Pediatrics, Cincinnati Children's Hospital Medical Center, Cincinnati, OH, 45229 USA

[2]Department of Pediatrics, University of Cincinnati, Cincinnati, OH, 45229, USA

[3]Division of Cardiology, Johns Hopkins Medical Institutions, Baltimore, MD 21205. USA

[4]Division of Cardiovascular Health and Disease, Department of Internal Medicine, University of Cincinnati, OH 45267, USA

[5]Myologica, LLC, New Market, MD 21774, USA

[6]Department of Orthopedics and Center for Biomedical Engineering and Technology (BioMET), University of Maryland School of Medicine, Baltimore, MD

Supplementary Figures 1-5

Supplementary Figure 1

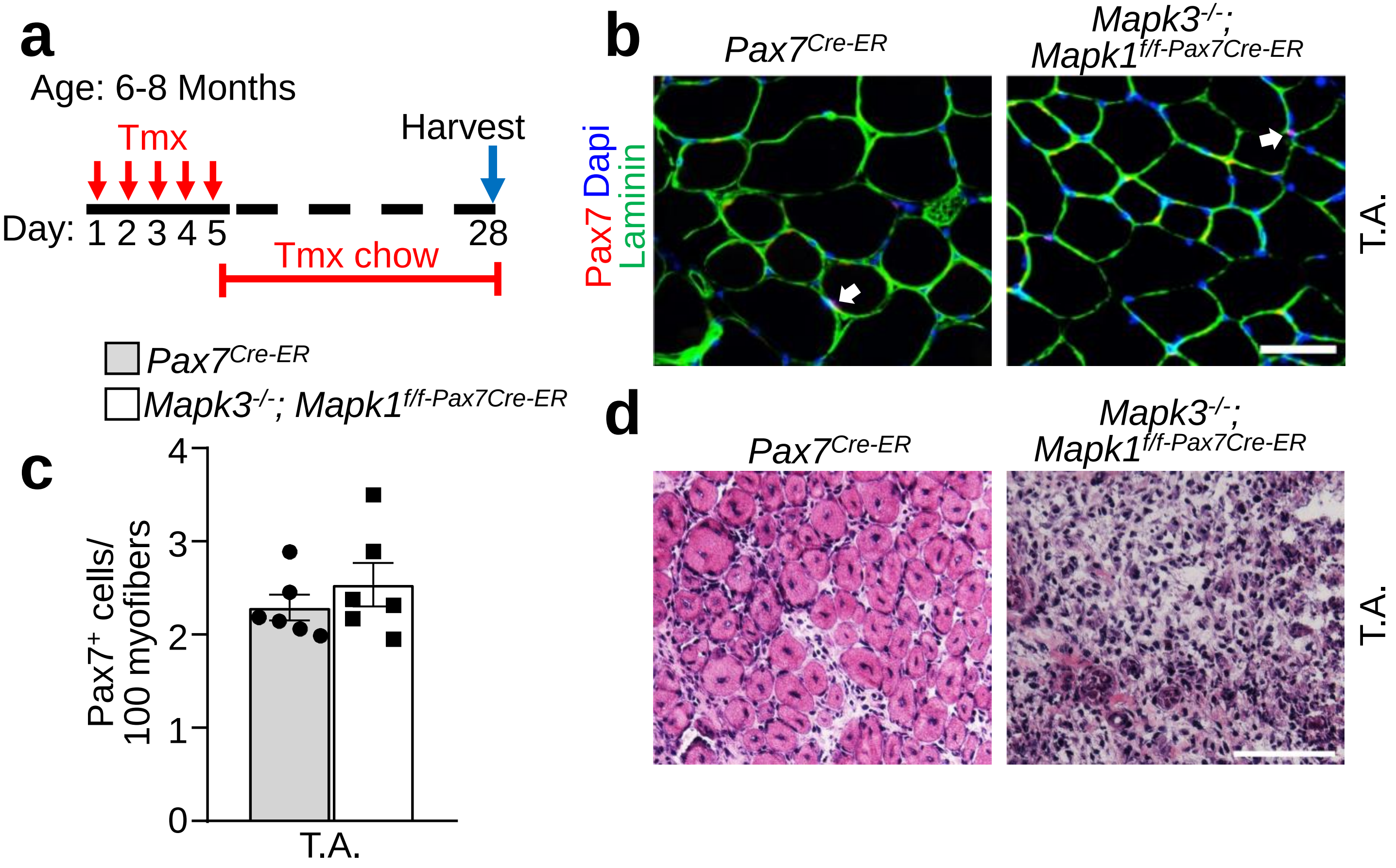

Supplementary Fig. 1. Normal satellite cells numbers in adult mice lacking *Mapk3* and *Mapk1*. a, Schematic representation of the tamoxifen (tmx) treatment regimen. Adult mice (6-8-month-old) received a daily tmx injection for 5 consecutive days and were subsequently placed on tmx chow after for 3 weeks. **b**, Representative immunostained sections of the tibialis anterior (TA) muscle for Pax7 (arrows) and laminin (green). Dapi stained nuclei are in blue. Scale bar = 50 μm. **c**, Quantification of Pax7$^+$ cells in TA muscle sections from mice of the indicated genotypes. n = 6 mice per group. Data represent mean ± SEM. **d**, Representative H&E-stained sections of the TA muscle 7 days post CTX injury. Scale bar = 100 μm. Samples from 3 different mice were analyzed.

Supplementary Figure 2

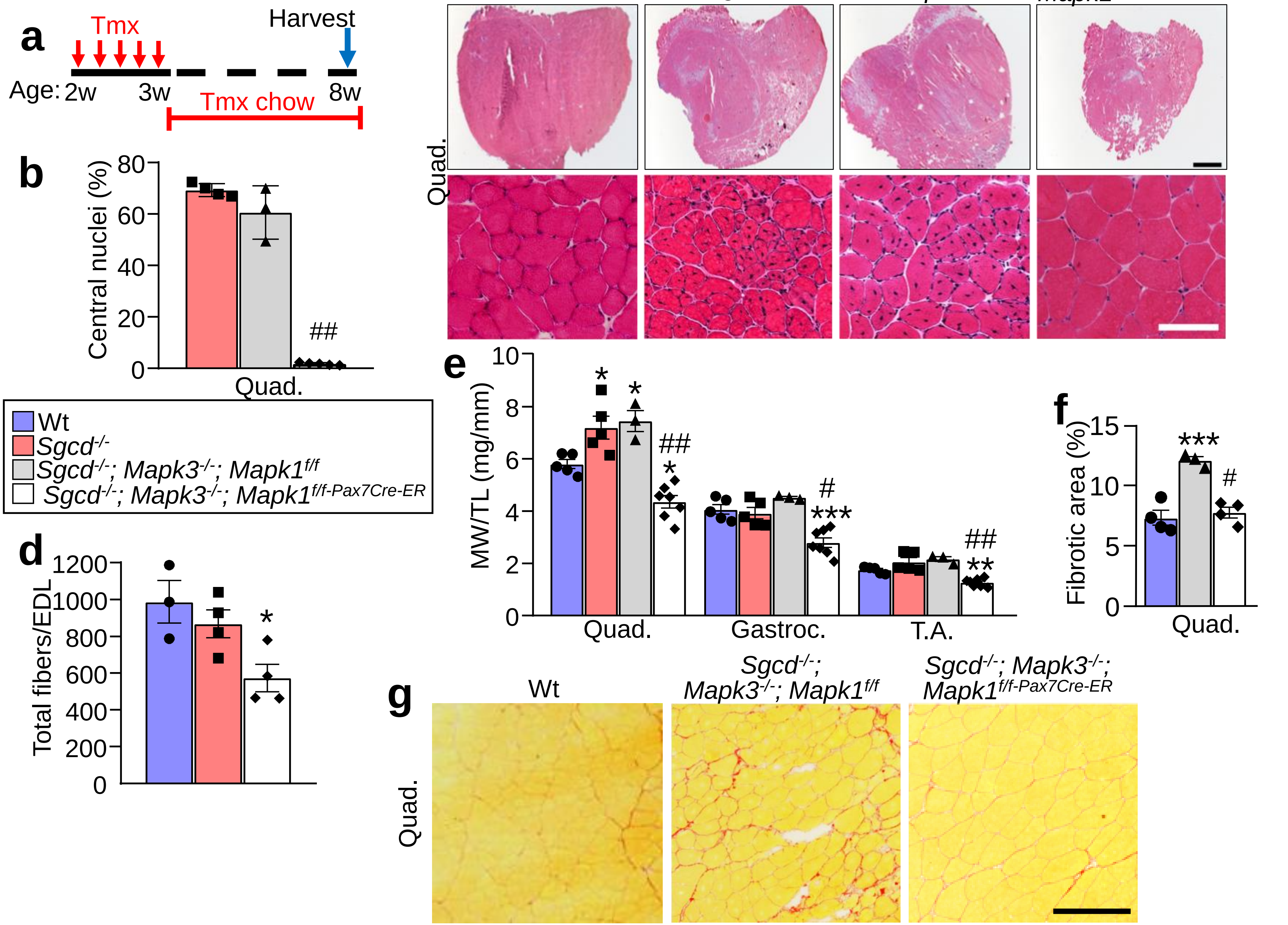

Supplementary Fig. 2. Satellite cell depletion prior to myofiber degeneration in *Sgcd*$^{-/-}$ mice.

a, Schematic of the tamoxifen (tmx) treatment. Two-week-old mice received a daily tmx injection for five consecutive days and were subsequently placed on tmx chow after weaning. **b**, Quantification of the number of myofibers with centrally located nuclei in quad muscle sections from mice of the indicated genotypes. n = 4 *Sgcd*$^{-/-}$ mice; n = 3 *Sgcd*$^{-/-}$; *Mapk3*$^{-/-}$; *Mapk1*$^{f/f}$ mice and n = 5 *Sgcd*$^{-/-}$; *Mapk3*$^{-/-}$; *Mapk1*$^{f/f\text{-}Pax7Cre\text{-}ER}$ mice. One-way ANOVA with Tukey's multiple comparisons test was used to determine significance, $^{\#\#}$P < 0.001 vs disease controls. **c**, Representative H&E-stained histological sections of the quad muscle from mice of the indicated genotypes. Scale bars = 100 µm. **d**, Quantification of the total myofibers present in the extensor digitorum longus (EDL) muscle from 2-month-old mice of the indicated genotypes. n = 3 Wt mice; n = 4 *Sgcd*$^{-/-}$; *Mapk3*$^{-/-}$; *Mapk1*$^{f/f}$ mice and n = 4 *Sgcd*$^{-/-}$; *Mapk3*$^{-/-}$; *Mapk1*$^{f/f\text{-}Pax7Cre\text{-}ER}$ mice. A 1-way ANOVA with Tukey's multiple comparisons test was used to determine significance, *P < 0.05 vs wt. **e**, Muscle weights (MW) normalized to tibia length (TL) from mice of the indicated genotypes at 2 months of age. n = 5 Wt mice; n = 5 *Sgcd*$^{-/-}$ mice, n = 3 *Sgcd*$^{-/-}$; *Mapk3*$^{-/-}$; *Mapk1*$^{f/f}$ mice and n = 7 *Sgcd*$^{-/-}$; *Mapk3*$^{-/-}$; *Mapk1*$^{f/f\text{-}Pax7Cre\text{-}ER}$ mice. A 1-way ANOVA with Tukey's multiple comparisons test was used to determine significance, ***P < 0.001 vs Wt, **P < 0.01 vs Wt, *P < 0.05 vs Wt, $^{\#}$P < 0.01 vs disease controls, $^{\#\#}$P < 0.001 vs disease controls. **f**, Quantification of the fibrosis present from images as shown in "**g**" from quad muscle sections from mice of the indicated genotypes. n = 4 Wt mice; n = 3 *Sgcd*$^{-/-}$; *Mapk3*$^{-/-}$; *Mapk1*$^{f/f}$ mice n = 4 *Sgcd*$^{-/-}$; *Mapk3*$^{-/-}$; *Mapk1*$^{f/f\text{-}Pax7Cre\text{-}ER}$ mice. One-way ANOVA with Tukey's multiple comparisons test was used to determine significance, ***P < 0.001 vs Wt, $^{\#}$P = 0.01 vs disease control. **g**, Representative Picrosirius Red-stained histological sections of the quad muscle from mice of the indicated genotypes as quantified in "**f**". Scale bar = 100 µm. Data represent mean ± SEM for all graphs.

Supplementary Figure 3

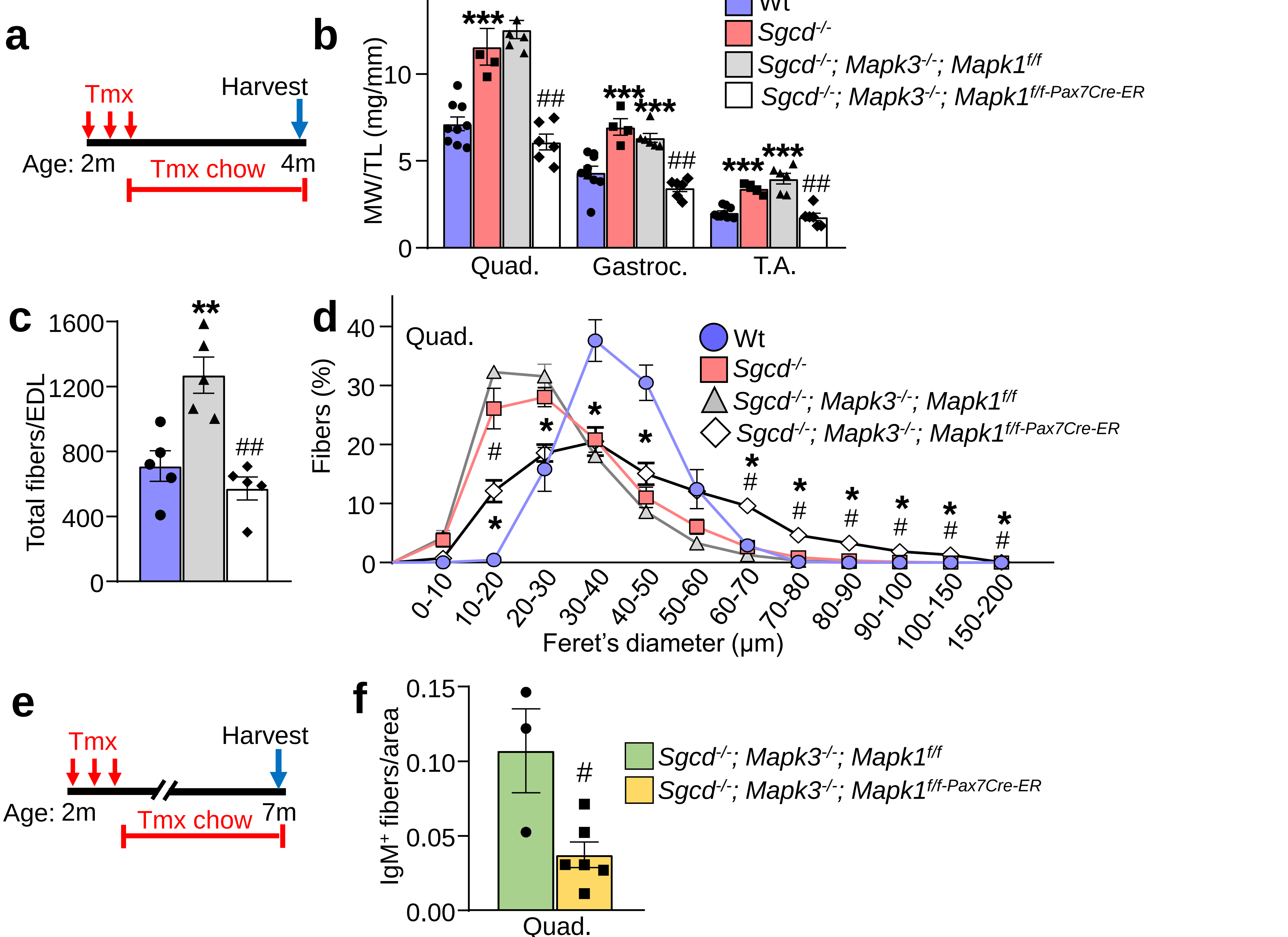

Supplementary Fig. 3. Satellite cell depletion in *Sgcd*$^{-/-}$ mice.

a, Schematic representation of the tamoxifen (tmx) treatment regimen. Two-month-old mice received a daily tmx injection for five consecutive days and were subsequently placed on tmx chow thereafter. **b**, Muscle weights (MW) normalized to tibia length (TL) from mice of the indicated genotypes at 4 months of age. n = 8 wt mice; n = 4 *Sgcd*$^{-/-}$ mice; n = 6 *Sgcd*$^{-/-}$; *Mapk3*$^{-/-}$; *Mapk1*$^{f/f}$ mice; n = 6 *Sgcd*$^{-/-}$; *Mapk3*$^{-/-}$; *Mapk1*$^{f/f\text{-}Pax7Cre\text{-}ER}$ mice. A 1-way ANOVA with Tukey's multiple comparisons test was used to determine significance, ***P < 0.001 vs Wt, $^{\#\#}$P < 0.001 vs disease controls. **c**, Quantification of the total myofibers present in the extensor digitorum longus (EDL) muscle from 4-month-old mice of the indicated genotypes. n = 5 mice for all genotypes. A 1-way ANOVA with Tukey's multiple comparisons test was used to determine significance, **P < 0.01 vs Wt, $^{\#\#}$P < 0.001 vs *Sgcd*$^{-/-}$; *Mapk3*$^{-/-}$; *Mapk1*$^{f/f}$. **d**, Minimal feret's myofiber diameter distribution from the quad muscle of 4-month-old mice with the indicated genotypes. n = 4 Wt mice; n = 4 *Sgcd*$^{-/-}$ mice; n = 3 *Sgcd*$^{-/-}$; *Mapk3*$^{-/-}$; *Mapk1*$^{f/f}$ mice and n = 3 *Sgcd*$^{-/-}$; *Mapk3*$^{-/-}$; *Mapk1*$^{f/f\text{-}Pax7Cre\text{-}ER}$ mice. A 1-way ANOVA with Tukey's multiple comparisons test was used to determine significance, *P < 0.05 vs Wt, $^{\#}$P < 0.05 versus disease controls. **e**, Schematic representation of the tmx treatment regimen. Two-month-old mice received a daily tmx injection for five consecutive days and were subsequently placed on tmx chow until 7 months of age. **f**, Quantification of immunoglobulin M (IgM) positive fibers in quad sections from mice of the indicated genotypes. n = 3 *Sgcd*$^{-/-}$*; Mapk3*$^{-/-}$*; Mapk1*$^{f/f}$ mice and n = 6 *Sgcd*$^{-/-}$*; Mapk3*$^{-/-}$*; Mapk1*$^{f/f\text{-}Pax7Cre\text{-}ER}$ mice. Significance was determined using a two-tailed Student's t-test, $^{\#}$P = 0.01. Data represent mean ± SEM for all graphs.

Supplementary Figure 4

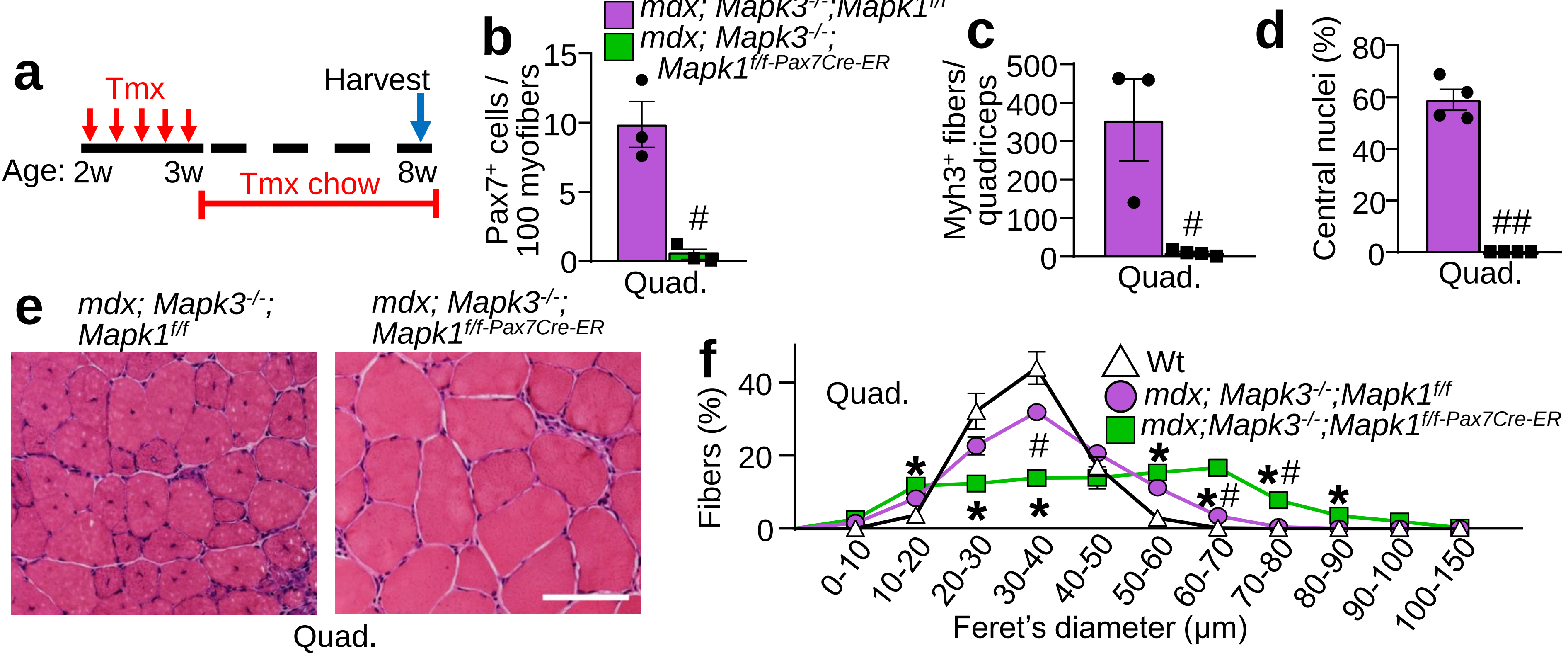

Supplementary Fig. 4. Improved histopathology in *mdx* mice lacking satellite cells.
a, Schematic representation of the tamoxifen (tmx) treatment in *mdx* mice. Two-week-old mice received a daily tmx injection for 5 consecutive days and were subsequently placed on tmx chow after weaning until 8 weeks of age. **b**, Quantification of Pax7$^+$ satellite cell numbers from quadriceps (Quad.) sections of 8-week-old mice of the indicated genotypes. n = 3 mice per group. Significance was determined using a two-tailed unpaired Student's t-test, $^{\#}P = 0.005$. **c**, Quantification of the number of Myh3 positive fibers in quad muscle sections of mice with the indicated genotypes. n = 3 *mdx*; *Mapk3$^{-/-}$*; *Mapk1$^{f/f}$* mice and n = 4 *mdx*; *Mapk3$^{-/-}$*; *Mapk1$^{f/f\text{-}Pax7Cre\text{-}ER}$* mice. Significance was determined using a two-tailed unpaired Student's t-test, $^{\#}P = 0.01$. **d**, Quantification of the number of myofibers with centrally located nuclei in quad muscle sections from mice of the indicated genotypes. n = 3 *mdx*; *Mapk3$^{-/-}$*; *Mapk1$^{f/f}$* mice and n = 4 *mdx*; *Mapk3$^{-/-}$*; *Mapk1$^{f/f\text{-}Pax7Cre\text{-}ER}$* mice. Significance was determined using a two-tailed unpaired Student's t-test, $^{\#\#}P < 0.001$. **e**, Representative H&E-stained histological sections of the quad muscle from mice of the indicated genotypes. Scale bar = 100 μm. **f**, Minimal feret's myofiber diameter distribution from the quad muscle of 2-month-old mice with the indicated genotypes. n = 4 mice per group. A 1-way ANOVA with Tukey's multiple comparisons test was used to determine significance, $^{*}P < 0.05$ vs Wt, $^{\#}P < 0.05$ vs *mdx*; *Mapk3$^{-/-}$*; *Mapk1$^{f/f}$*. Data represent mean ± SEM for all graphs.

Supplementary Figure 5

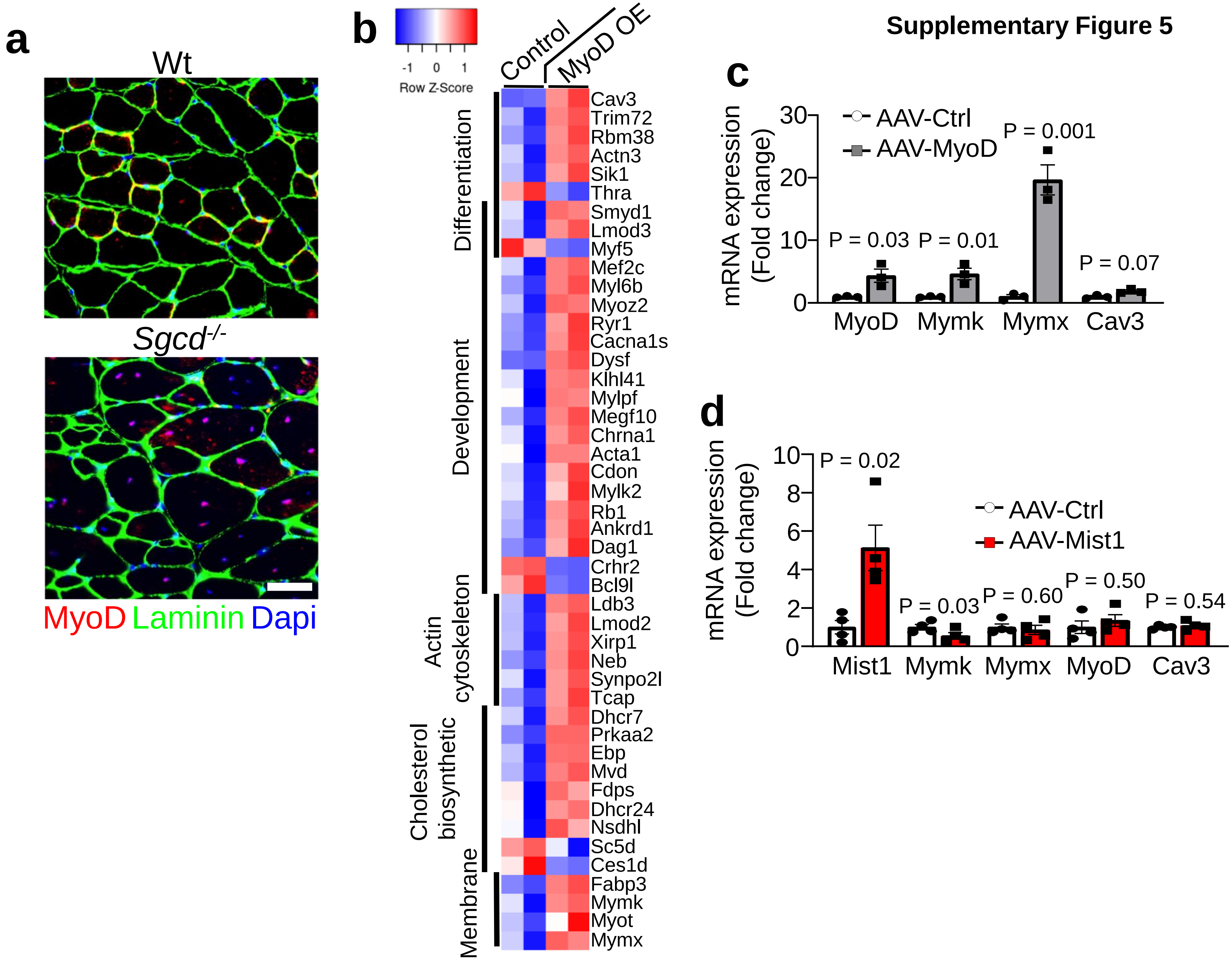

Supplementary Fig. 5. Analysis of the MyoD-directed muscle fetal gene program.
a, Representative tibialis anterior (TA) muscle sections immunostained for MyoD (red) and laminin (green) in wt and *Sgcd*$^{-/-}$ mice at 4 months of age. Dapi stained nuclei are in blue. Scale bar = 100 μm. Samples from 3 different mice were analyzed. **b**, Heatmap of selected up-regulated biological categories and dysregulated individual genes from C2C12 cells transduced with a doxycycline-inducible MyoD construct and differentiated for 3 days with or without dox (assayed with Affymetrix microarrays). Individual biological replicates are shown. **c,d**, mRNA gene expression analysis by qPCR for the indicated mRNAs from TA muscle of mice previously infected with AAV-MyoD, AAV-Ctrl or AAV-Mist1. n = 3 mice per group for **c** and n = 4 mice per group for **d**. Significance was determined using a two-tailed Student's t-test. Data represent mean ± SEM for all graphs. P values are indicated in panels c and d.
